# Tetracycline Resistance Genes Identified from Distinct Soil Environments in China by Functional Metagenomics

**DOI:** 10.3389/fmicb.2017.01406

**Published:** 2017-07-24

**Authors:** Shaochen Wang, Xia Gao, Yuejiao Gao, Yanqing Li, Mingming Cao, Zhenhua Xi, Lixing Zhao, Zhiyang Feng

**Affiliations:** ^1^College of Food Science and Technology, Nanjing Agricultural University Nanjing, China; ^2^Institute of Tibetan Plateau Research, Chinese Academy of Sciences Beijing, China; ^3^Yunnan Institute of Microbiology, Yunnan University Kunming, China

**Keywords:** functional metagenomics, soil, tetracycline resistance, efflux transporter, tetracycline destructase

## Abstract

Soil microbiota represents one of the ancient evolutionary origins of antibiotic resistance and has been increasingly recognized as a potentially vast unstudied reservoir of resistance genes with possibilities to exchange with pathogens. Tetracycline resistance is one of the most abundant antibiotic resistances that may transfer among clinical and commensal microorganisms. To investigate tetracycline resistance genes from soil bacteria in different habitats, we performed functional analysis of three metagenomic libraries derived from soil samples collected from Yunnan, Sichuan, and Tibet, respectively, in China. We found efflux transporter genes form all the libraries, including 21 major facilitator superfamily efflux pump genes and one multidrug and toxic compound extrusion (MATE) transporter gene. Interestingly, we also identified two tetracycline destructase genes, belonging to a newly described family of tetracycline-inactivating enzymes that scarcely observed in clinical pathogens, from the Tibet library. The inactivation activity of the putative enzyme was confirmed *in vitro* by biochemical analysis. Our results indicated that efflux pumps distributed predominantly across habitats. Meanwhile, the mechanism of enzymatic inactivation for tetracycline resistance should not be neglected and merits further investigation.

## Introduction

The widespread of antibiotic-resistant bacteria is an escalating global problem, underscoring the need for a larger arsenal of antibiotics and a further understanding of the ecology of the antibiotic “resistome”, including their origins, reservoirs and the underlying resistance mechanisms (Wright, 2007). It is now well accepted that resistance genes originated in environmental organisms long before the anthropogenic use of antibiotics (Forsberg et al., 2014; Perry and Wright, 2014; Perry et al., 2014; Graham, 2015). Growing evidences implicated that the resistance genes were transmitted between environmental bacteria and pathogens via horizontal gene transfer (Forsberg et al., 2012; Rolain, 2013). Besides, numerous resistance genes have been detected in different kinds of food (Aquilanti et al., 2007; Osimani et al., 2017), and the diet is one of the main route for the entrance of antibiotic resistance genes and bacteria within the humans (Wang et al., 2012; Rolain, 2013). Worse still, the exchange of antibiotic resistance genes has also been found from soil to food-producing animals (Wang et al., 2017). Thus, it emphasizes the important role of environmental bacteria as a vast reservoir of antibiotic resistance genes. It has also been suggested that these resistance genes in their original reservoirs may serve very different functions from the “weapon-shield” role they play in clinic. Therefore, the occurrence of these reserve genes that could express resistance function by “functional shift” may be greatly unexplored (Martinez, 2008). Vast majority of these unknown genes in environmental organisms are unable to be identified by traditional survey methods like PCR or microarray which generally based on known sequences. Functional metagenomics allows for the exploration of novel resistance genes, and is independent of culture and sequence bias (Allen et al., 2009; Sommer et al., 2009; Vercammen et al., 2013; Gibson et al., 2015).

The tetracyclines is one of the most widely used classes of broad spectrum antibiotics in clinic and agriculture due to their excellent therapeutic index, low cost, oral administration, and few side effects (Thaker et al., 2010). After over 60 years of extensive use of tetracyclines, the prevalence of tetracycline resistance has alarmingly increased, and become one of the most abundant antibiotic resistances among clinical and commensal microorganisms. To date, more than 50 different tetracycline resistance genes have been identified, conferring resistance primarily through three mechanisms: active efflux, ribosomal protection and enzymatic inactivation of the antibiotics. The first two mechanisms currently predominate in clinical settings (Roberts, 2005; Thaker et al., 2010). Due to the horizontal exchange of genes on mobile genetic elements such as plasmids and transposons, numerous tetracycline efflux, and ribosomal protection genes spread rapidly in a variety of species and ecosystems over time (Roberts, 2005; Knapp et al., 2010; Thaker et al., 2010). In contrast, enzyme inactivation, a common and preferred resistance mechanism of most natural-product antibiotics (Walsh, 2000), was far scarcely observed for tetracycline resistance. Only one enzyme, Tet(X), has been found in human pathogens with confirmed *in vitro* activity (Yang et al., 2004; Leski et al., 2013). However, a recent finding of a family of tetracycline destructases from soil metagenomic sources has brightened the importance of this long been neglected mechanism (Forsberg et al., 2015). The present study was directed toward investigating tetracycline resistances from distinct soil environments in China using functional metagenomics. The exploration of diverse resistance genes in environmental organisms and its mobilization to clinic would aid in our understanding of the evolution of the resistome as well as anticipation of the emergence of new resistance mechanisms in clinic.

## Materials and Methods

### Metagenomic Library Construction

Three metagenomic libraries were constructed using soil samples collected from distinct area of China (**Table 1**). All of the sampling sites have no known exposure to antibiotics. The construction of the Yunnan and Tibet libraries were performed following the published methods (Brady, 2007; Gu et al., 2015). In brief, 200 g of meshed soil was used to extract the environmental DNA (eDNA). The eDNA was then purified and blunt-ended (Epicentre, Charlotte, United States). The resulting eDNA was ligated with the pWEB-TNC vector (Epicentre, Charlotte, United States), packaged (Epicentre, Charlotte, United States) and transfected into *Escherichia coli* (*E. coli*) EPI100-T1^R^ (Epicentre, Charlotte, United States). The Sichuan library was constructed with pJTU2554 (Li et al., 2008) vector in the *E. coli* JTU007 (Zhou et al., 2012) host using the same methods.

**Table 1:** Cosmid libraries constructed with soil samples collected from distinct area of China.

Library name	Sampling site	Altitude (meters)	Vector	Vector resistance mark ^a^	Host	Average insert size (kb)	No. of clones	Gb of cloned DNA
Yunnan	Forest (N23°44′, E101°71′)	2100	pWEB-TNC	Chl, Amp	*E. coli* EPI100-T1^R^	38	1.28 × 10^7^	486
Tibet	Mount Qomolangma (N28°21′, E86°56′)	5500	pWEB-TNC	Chl, Amp	*E. coli* EPI100-T1^R^	38	1.04 × 10^7^	395
Sichuan	Forest (N29°34′, E103°20′)	900	pJTU2554	Apr	*E. coli* JTU007	30	4.80 × 10^4^	1.44

*^a^Chl, chloramphenicol; Amp, ampicillin; Apr, apramycin.*

### Screening of Tetracycline Resistant Clones

The tetracycline-resistant clones were selected as previously described (Allen et al., 2009). Five thousand individual clones of the metagenomic libraries were inoculated in 3 ml Luria–Bertani (LB) broth containing 50 μg/ml chloramphenicol and 100 μg/ml ampicillin (clones form Tibet and Yunnan libraries), or 50 μg/ml apramycin (clones from Sichuan library). After 2–4 h at 37°C with shaking, cultures were then plated at ∼5 × 10^5^ CFU/plate on LB agar plates containing 10 μg/ml tetracycline together with 50 μg/ml chloramphenicol or 50 μg/ml apramycin, according to the selection marker on the vectors. Plates were incubated at 37°C for up to 2 days, and then resistant colonies were picked for further study.

### Identification and Sequencing of Active Genes

The gene responsible for tetracycline resistance was identified by subcloning. The cosmid DNA from the resistant clone was partially digested with *Sau* 3AI (Takara Bio, Dalian, China), and DNA fragments of 2–3 kb were then recovered and ligated into *Bam* HI digested and alkaline phosphatase treated pTG19-T vector (Generay Biotech, Shanghai, China), electroporated into *E. coli* EPI100-T1^R^, and plated onto LB screening agar plates for resistant subclones. All screened subclones were verified by restriction digestion and retransformation to confirm the phenotypes. The resulting positive recombinant clones were sequenced by using T7 promoter and M13 primers at first, then by primer-walking sequencing.

Sequence analysis was carried out with the BLASTx program^1^. Amino acid alignment was performed with the ClustalW2.0 program and phylogenetic analysis (Neighbor-joining tree) was done with MEGA6.0 using neighbor-joining (500 bootstrap replicates).

### Tetracycline Susceptibility Testing

The susceptibilities of the positive subclones to tetracycline were tested using the standardized broth microdilution method. All tests were performed and results were evaluated according to the Clinical and Laboratory Standards Institute guidelines (CLSI) document CLSI M100-S24 (2014). Assays were performed in duplicate and experiments were conducted twice with *E. coli* ATCC 25922 and *E. coli* EPI100-T1^R^ carrying the empty pTG19-T vector as the reference strains.

### Construction of Expression Recombinant Plasmids

The full-length ORF encoding putative tetracycline destructases was amplified by PCR with the following primer pair: 1435-F (5′-ggaattccatatgtctgctacaaataaaattctcgt-3′)/1435-R (5′-ccgctcgagcttttcataatctggcaaagaaatgg-3′). *Nde* I or *Xho* I sites added for cloning are underlined. *Nde* I/*Xho* I-digested PCR product was cloned into *Nde* I/*Xho* I-digested pET30a(+) vector. The construct was then transformed into *E. coli* BL21 (DE3) for overexpression.

### Enzyme Expression and Purification

The putative tetracycline-inactivating enzyme was expressed and purified as previously described with some modification (Gu et al., 2015). Briefly, overnight culture was used to inoculate 500 ml kanamycin supplemented LB broth (50 μg/ml) (1:1000 dilution) and cultured at 37°C with shaking until the OD_600 nm_ value reached 0.6. The sample was then cooled to 18°C and 0.4 mM isopropyl β-D-1-thiogalactopyranoside (IPTG) was added to induce protein expression for an additional 18 h. Cells were harvested by centrifugation at 4°C, resuspended in lysis buffer (50 mM Tris-HCl [pH 8.0], 100 mM NaCl, 1% Triton X-100), and subsequently disrupted by sonication. The protein mixture was collected at 15,000 rpm for 15 min at 4°C. Proteins were purified via Ni-NTA resin (Bio-Rad, Hercules, United States). After washing with 10 column volumes of washing buffer (50 mM Tris-HCl [pH 8.0], 100 mM NaCl), the bound proteins were recovered with elution buffer (50 mM Tris-HCl [pH 8.0], 100 mM NaCl, 500 mM imidazole). The purified protein was analyzed by SDS-PAGE electrophoresis, flash frozen and stored at –80°C.

### *In Vitro* Enzymatic Reactions

*In vitro* enzymatic reactions were performed following the method previously described with a little modification (Forsberg et al., 2015). A total volume of 564 μL reaction mixture contained 1.4 mM tetracycline and 350 μg purified enzyme was prepared with a NADPH regenerating system. The reaction started with the addition of thawed enzyme. Then, 2000 μL quencher solution (equal parts methanol and 0.25 M HCl) was added to each reaction mixture at indicated time points. The enzymatic degradation products were extracted by ethyl acetate, and redissolved with 400 μL methanol for reverse phase HPLC analysis.

### Analysis of Tetracycline Degradation Products by HPLC

Products generated from enzymatic inactivation of tetracycline were analyzed by reverse phase HPLC (InertSustain^®^ C18, 4 × 250 mm, 5 μm) using a linear gradient of H_2_O:MeOH (20:80 to 80:20) over 20 min at 1 ml/min.

### Nucleotide Sequence Accession Numbers

All nucleotide sequences have been deposited in GenBank with the following accession numbers: KX161706–KX161713, KY697282–KY697295, and KY853665–KY853666.

## Results

### Construction of the Metagenomic Libraries

We collected three soil samples from distinct area of southwest China (**Table 1**). The sampling sites have different altitude and climate. DNA isolated from soils collected in Yunnan and Tibet was used to construct cosmid libraries that contained 12.8 and 10.4 million unique members, respectively. The average insert size of these two libraries was approximately 38 kb. Besides, a cosmid library containing about 4.80 × 10^4^ individual members was also constructed using eDNA extracted from Sichuan soil, with an average insert size of 30 kb.

### Screening of Tetracycline Resistant Clones and Susceptibility Assays

To identify tetracycline resistant clones, we subjected the metagenomic libraries to a functional selection on LB agar amended with tetracycline. In total, we obtained thirty clones that conferred resistance on *E. coli.* We determined the MICs of those thirty clones that confer reproducible decrease in susceptibility to tetracycline (**Table 2**). All of these clones showed increased resistance to tetracycline compared to the vector-only control. The overall MIC values ranged from 16 to 256 mg/ml, and 50% of the clones presented high-level resistance to tetracycline (MIC ≥ 8 × resistant breakpoint). The mean MIC values of the Yunnan and Tibet clones (58 and 65 mg/ml, respectively) were much higher than that of the Sichuan clones (32 mg/ml).

**Table 2:** Clones from three soil metagenomic libraries that confer tetracycline resistance on *Escherichia coli*.

Clone	Tetracycline MIC (μg/ml)	Mode of action	Similarity (organism)	Accession no. of closet match (% identity)
MQ203	32	Efflux pump	Tetracycline efflux MFS transporter (*Microbacterium sp*. Root280D1)	WP_056279670.1 (92)
MQ730	16	Efflux pump	Tetracycline efflux MFS transporter (*Microbacterium* sp. *CGR1*)	WP_053097549.1 (88)
MQ1411	64	Efflux pump	Tetracycline efflux MFS transporter (*Micrococcales bacterium* 72–143)	OJX66894.1 (78)
MQ1715	128	Efflux pump	Tetracycline efflux MFS transporter Tet(42) (*Micrococcus sp*. SMCC G887)	WP_063855882.1 (88)
MQ1987	64	Efflux pump	Tetracycline transporter (*Microbacterium sp*. Leaf320)	WP_056514290.1 (91)
MQ1629	32	Efflux pump	MATE efflux family protein (*Acidobacteria bacterium* OLB17)	KXK01510.1 (59)
MQ776	64	Enzyme inactivation	Tetracycline destructase Tet(47) (Uncultured bacterium)	AKQ05891.1 (73)
MQ1435	64	Enzyme inactivation	Tetracycline destructase Tet(47) (Uncultured bacterium)	AKQ05891.1 (80)
YN141	256	Efflux pump	MFS transporter (*Streptomyces griseus*)	WP_044369073.1 (78)
YN623	32	Efflux pump	MFS transporter (*Labrys* sp. WJW)	WP_068282769.1 (80)
YN629	32	Efflux pump	MFS transporter, EmrB/QacA subfamily (*Nocardioides terrae*)	SFC16744.1 (86)
YN652	32	Efflux pump	MFS transporter (*Labrys* sp. WJW)	WP_068282769.1 (86)
YN1039	64	Efflux pump	Tetracycline resistance MFS efflux pump [*Devosia sp*. 66-22]	OJX53909.1 (80)
YN1046	64	Efflux pump	Tetracycline resistance MFS efflux pump [*Streptomyces griseus*]	WP_044369073.1 (78)
YN1332	64	Efflux pump	MFS transporter, EmrB/QacA subfamily (*Nocardioides lianchengensis*)	SDD59586.1 (88)
YN1338	128	Efflux pump	MFS transporter (*Aeromicrobium* sp. Root344)	WP_056210997.1 (85)
YN1469	32	Efflux pump	MFS transporter Tet (G) (*Burkholderia* sp. BT03)	WP_007744101.1 (98)
YN1865	32	Efflux pump	MFS efflux pump (*Streptomyces* sp. LUP30)	WP_069766755.1 (90)
YN2297	64	Efflux pump	MFS efflux pump Tet (A) family (*Pelomonas* sp. Root1237)	WP_056192001.1 (92)
YN2306	64	Efflux pump	MFS efflux pump Tet (A) family (*Pelomonas* sp. Root1237)	WP_056192001.1 (89)
YN2386	16	Efflux pump	MFS efflux pump (*Gordonia soli*)	WP_007619561.1 (81)
YN2560	32	Efflux pump	MFS transporter, DHA1 family, bicyclomycin/chloramphenicol resistance protein (*Dyella* sp. OK004)	SFS19133.1 (82)
SC6	32	Efflux pump	MFS efflux pump Tet (A) family (*Pelomonas* sp. Root1237)	WP_056192001.1 (89)
SC2451	32	Efflux pump	MFS transporter Tet(G) (*Burkholderia* sp. BT03)	WP_007744101.1 (99)

### Identification of Tetracycline Resistance Genes and Phylogenetic Analysis

**FIGURE 1:**
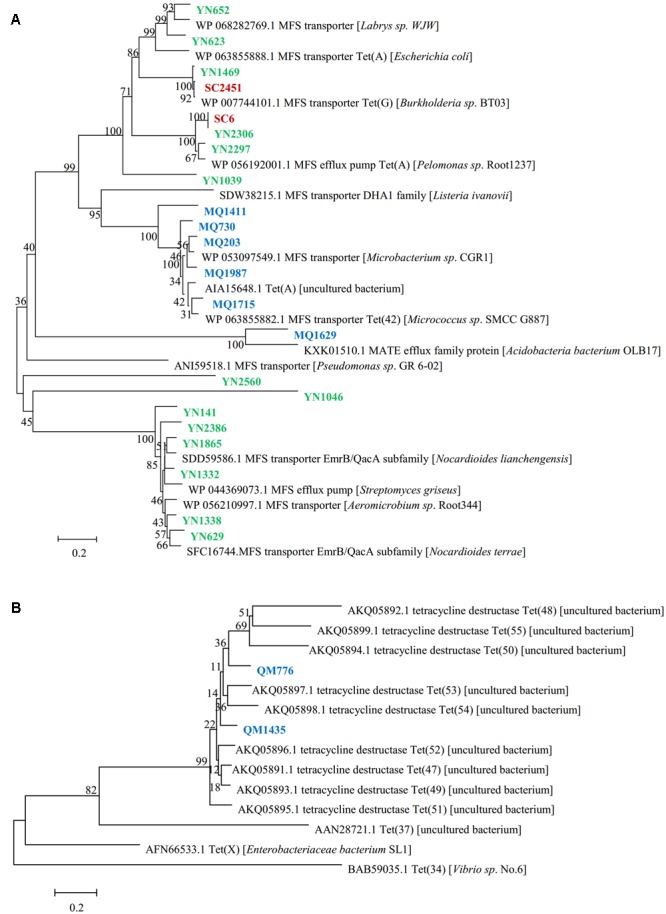
Phylogenetic tree inferred with neighbor-joining for the predicted amino acid sequences of **(A)** tetracycline resistance transporter, **(B)** tetracycline destructase. Tetracycline resistant clones from Mount. Qomolangma soil metagenomic library are shown in blue, clones from Yunnan library are colored in green and clones from Sichuan library are colored in red.

The genes responsible for tetracycline resistance were identified by sequencing and BLAST analyzing the resistant subclones, and 24 unique resistance genes of different types were obtained (**Table 2**). Of these functional genes, 22 showed homology to previously reported tetracycline transporters, including twenty one major facilitator superfamily (MFS) transporters and one multidrug and toxic compound extrusion (MATE) family efflux. MFS transporter genes were obtained in all of the three libraries screened in this study, suggesting a wide distribution of this gene family across the area and diverse soils. The deduced amino acid sequences of them showed a moderate similarity with one another (average pairwise amino acid identity 47.3% ± 7.3%). The two Sichuan-derived MFS transporters, SC6 and SC2451, shared the same amino acid sequences with YN2306 and YN1469 from Yunnan library, respectively, implying a horizontal transfer potential of this element. In contrast, the amino acid sequences of MFS transporters from Tibet were divergent from that of Yunnan-derived ones, and therefore clustered separately in the phylogenetic tree (**Figure 1A**). From Tibet library, we also identified a putative MATE efflux protein. BLAST analysis of the putative MQ1629 protein yielded the best match with a MATE efflux from Acidobacteria bacterium OLB17 (KXK01510.1, 59%). The MATE family efflux was reported to confer resistance to a wide spectrum of cationic toxic agents such as organic acids, plant hormones and secondary metabolites (Li and Nikaido, 2009). To evaluate the activity of this functional MATE transporter gene, we additionally determined the antimicrobial susceptibility of clone MQ1629 for ceftazidime, gentamycin, ciprofloxacin. However surprisingly, this putative efflux conferred resistance only to tetracycline (32 μg/ml) (data not shown).

In addition, we also identified two functional genes showed homology to a novel family of tetracycline destructase newly identified from soil metagenomes (Forsberg et al., 2015; **Figure 1B**). The two deduced proteins we reported here, sharing 73% amino acid identity with each other, harbored an average identity of 61.6% with those nine previously found tetracycline destructases. But they showed low identities of 20 and 21%, respectively, to Tet(X), the only one confirmed tetracycline inactivation enzyme from clinical bacterium.

### *In Vitro* Enzymatic Inactivation

**FIGURE 2:**
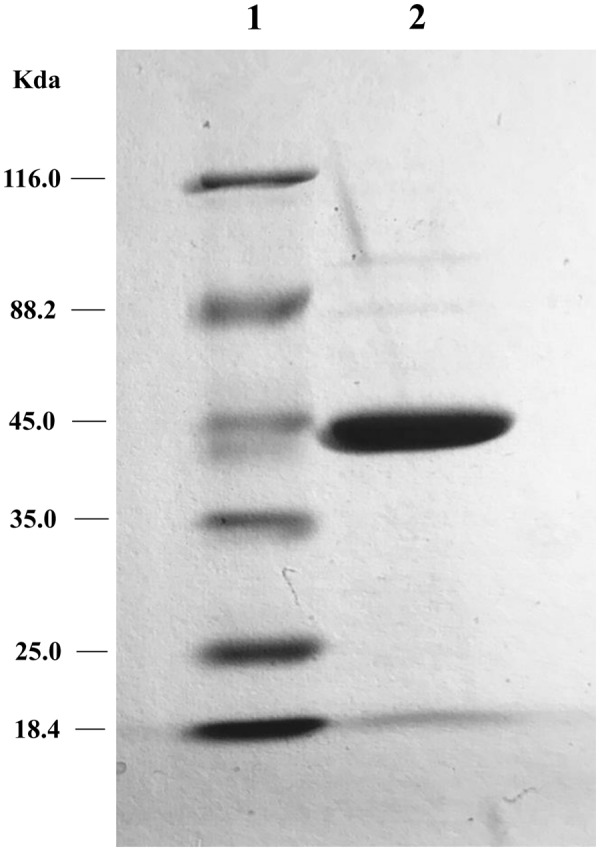
SDS-PAGE gel electrophoresis of the tetracycline inactivation enzyme. Lane 1: protein molecular weight marker; lane 2: purified protein of MQ1435.

**FIGURE 3:**
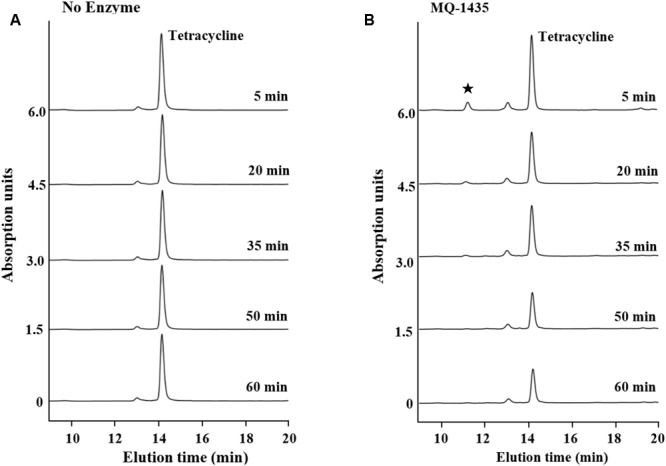
Reverse-phase PHLC separation of tetracycline and enzymatically catalyzed degradation product at 260 nm. **(A)** Reaction with no enzyme; **(B)** Reaction with the purified enzyme of MQ-1435. ^★^indicates the unstable degradation product.

The two putative tetracycline destructases we found shared high amino acid identity with each other and the same MIC to tetracycline (64 μg/ml). We deduced that the inactivation mechanism of these two enzymes may be the same, so MQ1435 clone was chosen for further study. The relative molecular mass of the putative MQ1435 enzyme was estimated to be 43 kDa by SDS-PAGE analysis (**Figure 2**). Reverse-phase HPLC was used to monitor the progression of each inactivation reaction. The result indicated that the tetracycline substrate eliminated with time (**Figure 3B**), however, no obvious new stable product was observed during the *in vitro* inactivation reaction. For control reaction that performed without enzyme, no obvious change in tetracycline substrate was generated (**Figure 3A**).

## Discussion

Using a functional metagenomics approach, we identified 21 MFS transporter genes from all three soil libraries. The Tibet-derived MFS transporters showed higher sequence similarity from each other, and clustered separately from those derived from the other two libraries. In addition, sequence and phylogenetic analysis showed that they were more homologous to the deduced MFS transporters obtained from sequenced metagenomes than those derived from cultured bacterium. The MATE transporter and inactivation enzyme genes were only found from Tibet library. These distinctions may be due to the specificity of the sampling site of this library. The soil was sampled at 5500 m altitude of Mount Qomolangma and there was no known exposure to antibiotics at the sampling point. Soil resistome composition has been demonstrated to be closely associated with different soil types and microbial taxonomic structures (Forsberg et al., 2014). Our results verified this correlation to some extent, and implied that the microbe derived from pristine environments could be a potential reservoir for discovering novel resistance genes, considering that the clinical resistome driven by antibiotics induced selection pressure is typically encoded by a much smaller pool of circulating resistance genotypes compared to the vast diverse environmental resistome (Davies and Davies, 2010; Martinez et al., 2015).

Drug inactivation, a common mechanism of pathogens to resist most naturally derived antibiotics such as the aminoglycosides and β-lactams (Walsh, 2000), was far scarcely observed for tetracycline. *tet*(X) was the first tetracycline inactivation gene identified early in 1988 from *Bacteroides fragilis* (Park and Levy, 1988). It has been demonstrated that Tet(X) is a flavin-dependent monooxygenase that inactivates tetracycline by monohydroxylation followed by non-enzymatic decomposition of unstable product. Later, another two predicted tetracycline inactivation genes, *tet*(34) and *tet*(37), were identified from *Vibrio sp*. no. 6 isolated from intestinal contents and an oral metagenome, respectively (Nonaka and Suzuki, 2002; Diaz-Torres et al., 2003). Strikingly, *tet*(X) was the only tetracycline inactivation gene that have been reported in human pathogens to date (Leski et al., 2013). Therefore, tetracycline inactivation has long been considered as a rare mechanism for tetracycline resistance. However, Forsberg et al. (2015) recently discovered nine tetracycline destructase genes from agricultural and grassland soil metagenomes, although three of them showed no *in vitro* activity. Here, we further identified two putative inactivation enzyme genes from a remote soil collected from Mount Qomolangma. Previously study with Tet(X) indicated that enzymatic degradation products of tetracyclines are stable at low pH (Yang et al., 2004; Moore et al., 2005). Similar with the results observed for the five of six functional destructases described by Forsberg et al. (2015), no obvious signatures of new stable product was detected in our study, indicating that these enzymes might inactivate tetracycline via diverse oxidative mechanisms different from Tet(X). Although the exact degradation mechanism of the enzyme we found remains unclear, our findings confirm that tetracycline inactivation genes may be much more widely distributed in natural soil habitats than previously recognized, and obviously brighten the importance of this long been neglected mechanism. Nevertheless, further work is needed to figure out how pH condition affects the stability of the degradation product of our enzyme, as well as to gain clear understanding of the degradation mechanism.

Functional metagenomic approach is able to reveal novel resistance genes that are unrecognizable as resistance genes on the basis of sequence from the vast environmental resistome (Allen et al., 2009; Gibson et al., 2015). Further, given that all the genes yielded by this approach are functional in *E. coli*, they clearly have the potential to be transferred and functional in pathogens. If there is a barrier to gene transfer between environmental microbes and pathogens, it is not due to functional compatibility. Several studies have revealed that the exchange of antibiotic resistance genes between bacteria from soils/animals and clinical pathogens occurred via horizontal gene transfer, and the frequency of gene exchange might be greatly underestimated (Smillie et al., 2011; Forsberg et al., 2012; Liu et al., 2016; Ma et al., 2016). Worse still, resistance genes residing in the abundant environmental reservoir were thought to have a larger threat for entering the circulating pathogenic resistome (Davies and Davies, 2010; Martinez et al., 2015), emphasizing the clinical importance of the environmental resistome. Consequently, further exploration of the diverse resistome in extend environments is critical and essential for a deeper understanding of the antibiotic resistance from an ecological perspective.

In this study, through functional metagenomic selection, we discovered several tetracycline resistance genes of different classes from soil metagenomes. Efflux pump genes were found to be predominant distributed across soil types and habitats, while two enzymatic inactivation genes were also identified. The tetracycline degradation activity of the expressed enzyme was confirmed *in vitro*. The results demonstrate that the enzymatic inactivation mechanism for tetracycline resistance should not be neglected and merits further investigation to figure out the diverse degradation mechanisms. Besides, it is necessary and urgent to monitor their spread in clinical settings.

## Author Contributions

SW, XG, and ZF designed research; SW, XG, YG, YL, MC, ZX, and LZ performed research; SW, XG, and ZF analyzed data; WS and ZF wrote the paper.

## Conflict of Interest Statement

The authors declare that the research was conducted in the absence of any commercial or financial relationships that could be construed as a potential conflict of interest.

## Footnotes

1 http://blast.ncbi.nlm.nih.gov
